# The application of rapid evaporative ionization mass spectrometry in the analysis of *Drosophila* species—a potential new tool in entomology

**DOI:** 10.1098/rsob.200196

**Published:** 2020-11-25

**Authors:** Iris Wagner, Natalie I. Koch, Joscelyn Sarsby, Nicola White, Tom A. R. Price, Sam Jones, Jane L. Hurst, Robert J. Beynon

**Affiliations:** 1 Centre for Proteome Research, Institute of Systems, Molecular and Integrative Biology, University of Liverpool, Crown Street, Liverpool L69 7ZB, UK; 2 Ecology and Evolution Group, Institute of Infection, Veterinary and Ecological Sciences, University of Liverpool, Crown Street, Liverpool L69 7ZB, UK; 3 International Pheromone Systems Ltd, Unit 8, West Float Industrial Estate, Millbrook Road, Wallasey, Wirral CH41 1FL, UK; 4 Mammalian Behaviour and Evolution Group, Institute of Infection, Veterinary and Ecological Sciences, University of Liverpool, Leahurst Campus, Neston CH64 7TE, UK

**Keywords:** REIMS, mass spectrometry, species identification, insects, *Drosophila*

## Abstract

There is increasing emphasis on the use of new analytical approaches in subject analysis and classification, particularly in respect to minimal sample preparation. Here, we demonstrate that rapid evaporative ionization mass spectrometry (REIMS), a method that captures metabolite mass spectra after rapid combustive degradation of an intact biological specimen, generates informative mass spectra from several arthropods, and more specifically, is capable of discerning differences between species and sex of several adult *Drosophila* species. A model including five *Drosophila* species, built using pattern recognition, achieves high correct classification rates (over 90%) using test datasets and is able to resolve closely related species. The ease of discrimination of male and female specimens also demonstrates that sex-specific differences reside in the REIMS metabolite patterns, whether analysed across all five species or specifically for *D. melanogaster.* Further, the same approach can correctly discriminate and assign *Drosophila* species at the larval stage, where these are morphologically highly similar or identical. REIMS offers a novel approach to insect typing and analysis, requiring a few seconds of data acquisition per sample and has considerable potential as a new tool for the field biologist.

## 1. Background

Insect identification and monitoring are essential to a number of diverse fields and settings, seeking to identify and study insect populations to learn more about their place in ecosystems as well as their impact on the environment and other species [1]. Long-term biodiversity and environmental impact studies [2,3] tend to observe and log the changes and make-up of insect populations. In other circumstances, such as biological control in pest management, maintaining the population of certain species is desirable or even necessary to sustain ecosystem balance [4]. Conversely, many arthropod species can cause considerable harm, economically as well as environmentally, and pose a risk to human health, requiring population control or reduction. Every year insect pests cause massive economic damage in agriculture and forestry [5,6], either by directly attacking important crops or through the transmission of diseases [7–11]. Biosecurity, which aims at curtailing risk through ‘biological harm’ [12], relies largely on rapid and accurate species identification as it affects risk assessments, the handling of imported goods and plans for future surveillance or eradication [13,14]. Correct identification likewise influences biological pest control strategies, such as the use of insect pheromones or prey/predator interactions, as their success is based on species-specific mechanisms [15–18]. In countries and regions where insects are a public health concern (for example, mosquitoes), specimens are routinely trapped for identification and other analytical purposes. Known vectors for diseases like malaria, dengue fever or Zika are monitored to inform authorities and the general public about threat levels and to predict disease transmission.

The long-established approach to identifying specimens is by morphological taxonomy, which uses taxonomic keys and requires or at least greatly benefits from experience. However, far more trained taxonomic experts are needed for diagnostics than are available to cover the range of programmes where species identification plays a pivotal role [19–22]. Additionally, not all insect specimens can be readily identified based on morphological characteristics. Existing morpho-taxonomic keys display deficiencies and limitations, especially when it comes to morphologically indistinguishable species, immature life stages, cryptic species or damaged specimens [23–25].

Increasingly, molecular analytical tools have been developed and applied to aid morphological examination and expand capabilities. These include cuticular hydrocarbon analysis [26], immunological [27] or protein-based assays [28] as well as mass spectrometry-based applications such as matrix-assisted laser desorption ionization mass spectrometry (MALDI-MS) [29]. However, DNA barcoding is often the method of choice, as it can handle a variety of sample conditions, developmental stages and cover a large number of species and taxa [30–32]. In routine identification or monitoring actions, identifying unknowns is not the only challenge. The large number of samples being collected requires fast processing, which has led to a number of automation efforts, most recently supported by machine learning and neural network algorithms [33–36].

New, easy-to-use high throughput tools capable of handling a variety of samples in vast amounts are still sought after and could provide much-needed support in the wide array of fields requiring rapid insect identification. Here, we introduce the use of rapid evaporative ionization mass spectrometry (REIMS) as an addition to the insect identification armamentarium. REIMS uses an ambient ionization source, specifically designed to analyse aerosols resulting from thermal disintegration caused by the passage of electricity through the sample of interest. The electric current is applied through diathermy tools and the resulting aerosol evacuated through a tube to the source and subsequently the mass spectrometer. Identification of single molecules from the acquired mass spectra is rarely the objective; instead pattern recognition is applied to identify unique mass patterns that facilitate classification and consequently sample identification. REIMS is a novel ionization technique, which has been developed to distinguish cancerous from healthy tissue during cancer surgery (iKnife) [37,38], but has found application in a variety of fields from food security and adulteration detection [39,40] to identification and characterization of bacterial strains [41–43] and, most recently, to recover information from rodent and human faecal matter [44,45].

A mixture of wild-trapped arthropod species and five laboratory-raised *Drosophila* species were used for a proof-of-principle study to investigate REIMS suitability for insect analysis and gauge its potential as an identification device. Our results demonstrate the techniques ability to distinguish species as well as the sex of specimens using models developed from the uninterpreted mass spectra that are derived from aerosol analysis.

## 2. Material and methods

### 2.1. Laboratory-raised *Drosophila*

For the laboratory-derived samples, *Drosophila melanogaster* (Dahomey), *D. simulans, D. subobscura*, *D. bifasciata*, *D. pseudoobscura* and *D. hydei* were reared in 250 ml glass bottles. All species were reared on standard ASG food (for 1 l of water: 10 g of agar, 20 g of yeast, 85 g of sugar, 60 g of cornmeal and 25 ml of nipagin (100 g l^−1^) except for *D. hydei* which was reared on banana food (for 1 l of water: 15 g agar, 30 g yeast, 150 g frozen bananas, 50 g blackstrap molasses, 30 g malt, 25 ml nipagin (100 g l^−1^). Species were reared at the optimal temperature according to their natural habitats; 25°C for *D. melanogaster, D. simulans* and *D. hydei*, 22°C for *D. pseudoobscura*, and 18°C for *D. bifasciata* and *D. subobscura* with a 12 L : 12 D cycle. Stocks were transferred to new food weekly, with adults replaced every four to five weeks. To represent what would realistically be collected in the wild, individuals for experiments were chosen at random, irrespective of age or virginity. Sex was determined under CO_2_ anaesthesia.

Species identity was checked using the mitochondrial universal barcode gene cytochrome oxidase subunit 1 (COI). DNA was extracted from three male flies with DNeasy kits (Qiagen) following the Qiagen invertebrate protocol. A sequence from COI was PCR amplified using the primers C1-J-1718 (5′–GGAGGATTTGGAAATTGATTAGT–3′) and C1-N-2191 (5′–CCCGGTAAAATTAAAATATAAACTTC–3′) using HotStart Taq (Promega) with (5 min initial heating, 30 cycles at 95°C for 30 s, 56 for 30 s and 72°C for 30, with a final elongation step of 72°C for 120 s). The products of these PCRs were visualized using SYBRSafe-stained gel electrophoresis. Products were then cleaned up using Exonuclease I and Shrimp Alkaline Phosphatase incubation using the recommended BioLine protocol. BigDye-based sequence reactions were carried out with both forward and reverse primers, followed by NaOH and ethanol clean-up and precipitation. Sequences were then analysed with an ABi 3500XL Genetic Analyser. Forward and reverse sequences for each species were aligned to derive a consensus sequence. The sequences were assessed using publicly available CO1 sequences from the same species available on the BOLD database.

### 2.2. Sample specimen collection and storage

For the initial study, a few individuals of five different arthropod species were collected from the University Leahurst campus, killed by freezing and stored at −20°C for 6 days. A total of 800 specimens of the *Drosophila* species *D. melanogaster*, *D. subobscura*, *D. pseudoobscura*, *D. bifasciata* and *D. simulans* were selected for REIMS analysis. The conspecifics of each species were separated into male and female subgroups to facilitate species as well as sex separation experiments. All specimens had been raised to their adult stage; further age differences as well as reproductive state were not taken into account. Specimens were directly transferred to fresh container vials and killed by freezing and stored at −20°C for 3–6 days, as samples were analysed over several days. Approximately 30 min prior to REIMS analysis, specimens were returned to room temperature. In a separate experiment, 3rd instar wandering stage larvae of *D. melanogaster* and *D. hydei* were collected, frozen, stored and returned to room temperature for REIMS as per the adults.

### 2.3. Rapid evaporative ionization mass spectrometry analysis

Samples were analysed via a rapid evaporative source (REIMS, Waters, Wilmslow, UK) attached to a Synapt G2Si instrument ion mobility equipped quadrupole time of flight mass spectrometer (Waters, UK). The specimens were burned/evaporated using a monopolar electrosurgical pencil (Erbe Medical UK Ltd, Leeds), which was connected to a VIO 50 C electrosurgical generator, providing electrical current, and to the source inlet via plastic tubing. A black rubber mat, placed underneath the samples, acted as a counter electrode and facilitated the flow of electric current. To avoid inhalation of fumes during analysis, the burning process was performed within a fume box (Air Science). Insects were analysed using a 40 W setting on the generator and the cutting option of the pencil. To increase conductivity and protect the counter electrode during analysis, specimens were placed on a piece of glass microfibre paper (GFP, GE Healthcare Whatman) on top of a wet paper surface (moistened with MilliQ water).

While burning the entire biomass of single specimens, the aerosol was aspirated through the pencil and the attached 3 m long tubing into the REIMS source, using a nitrogen powered venturi valve on the source inlet. To increase the aerosol capture of *Drosophila* species, a wide bore piece of plastic tubing was additionally placed over the tip of the electrosurgical pencil. A whistle incorporated into the Venturi tube guided the aerosol as well as a lock mass solution of leucine enkephalin (Waters, UK) in propan-2-ol (CHROMASOLV, Honeywell Riedel-de-Haën) into the source. This also filters the incoming aerosol to prevent larger particles from entering the inlet capillary. Inside the source, the ionized particles were declustered through contact with a heated impactor (Kanthal metal coil at 900°C).

Acquisition of the mass spectra was performed in negative ion mode at a rate of 1 scan per second over a mass/charge range of *m/z* 50–1200. The sample cone and heater bias were set to 60 V. Instrument calibration was performed daily in resolution mode using a 0.5 mM solution of sodium formate (flow rate 50 µl min^−1^). The lock mass solution (0.4 µg ml^−1^) was continuously introduced during sample analysis at a flow rate of either 50 µl min^−1^, used for the initial arthropod sample set, or 30 µl min^−1^, used for all *Drosophila* samples. For the first arthropod study, specimens were analysed in species order. All 800 *Drosophila* samples, as well as the *Drosophila* larvae, however, were analysed in a random order over 3 days.

### 2.4. Data analysis

The mass spectra were imported into the model building software packages; Offline Model Builder (OMB-1.1.28; Waters Research Centre, Hungary) and LiveID (Waters, UK), which allow separation of sample groups (classifications) based on principal component analysis (PCA) and linear discriminant analysis (LDA). Data were additionally analysed using R (version 3.6.1) [46] and the R Studio environment [47], by PCA and LDA, as well as random forest analysis.

For Offline Model Builder, the burn events of the analysed specimens were defined individually, summing up the MS scans within each chosen area. The option to create only a single burn event per sample was selected. Other pre-processing parameters included the intensity threshold, at 4 × 10^5^, spectra correction using the lock mass (leucine enkephalin, *m/z* 554.26) and background subtraction. To reduce the complexity of the mass spectral data, all acquired data points from *m/z* 50 to 1200 were combined into mass bins, each 0.1 *m/z* units wide. The subsequent model calculation was based on PCA-LDA. For LiveID, the data files were pre-processed using Progenesis Bridge (part of MassLynx software, Waters, UK): mass spectra were lock mass corrected, the background-subtracted and the scans summed and averaged to provide uniform burn events. This prevented incorrect splitting of burn events during the automated recognition in LiveID. Again, a mass range of *m/z* 50–1200 and a bin size of 0.1 were used to build models based on PCA and LDA.

The models built by Offline Model Builder and LiveID were cross-validated (leaving out 20% of data, for outliers the standard deviation multiplier was set to 5) to obtain the correct classification rate, as well as the number of failures and outliers and a matrix displaying the number of correctly and incorrectly identified samples of each classification. To additionally test obtained separation results, sample classifications were randomized and re-analysed, expecting a random distribution of samples and failed separation.

For further analysis with R, the data matrix of each model was exported as a .csv file from Offline Model Builder, containing information about classification and the normalised intensities for every mass bin. The matrices were used to perform random forest analysis in R using the package ‘randomForest' [48]. The datasets were randomly split into a training set (approx. 70% of the data) and a test set (approx. 30% of the data). Random forest results are displayed in the form of confusion matrices. Trees were conducted 10 times for every model (using a different, randomly selected subset of samples for training and testing every time); the numbers of correctly identified and confused samples were turned into percentages and averaged. The optimal number of trees and *mtry* value were determined during the first analysis of each model and kept the same for each repeated analysis. The numbers of trees and *mtry* values used for random forest analysis of the species and sex datasets are compiled in electronic supplementary material, figure S4. A second R package, called ‘randomForestExplainer' [49], was used to identify the most informative bins/ions that were driving class separation. For the sex separation results, PCA-LDA was also performed with R and plots created using ‘ggplot2’ [50].

All raw data files are freely available in the MetaboLights database with the accession number MTBLS1878 [51].

## 3. Results and discussion

**Figure 1.**
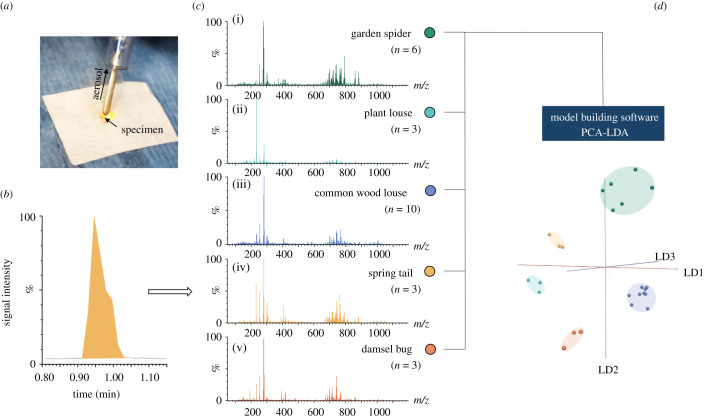
REIMS analysis of different arthropod species. Arthropods, killed by freezing, were analysed by REIMS using an electrosurgical pen with the knife attachment (*a*). Each sample from five different arthropod species was burned completely with little or no residual biomass in a burn event of about 10 s duration (*b*). The aerosol was aspirated and transported via a long tubing to the REIMS source attached to the mass spectrometer. There are recurrent differences between the acquired mass spectra of the different species, making them visually distinctive (*c*). High-resolution mass spectra were processed and analysed by PCA-LDA using the software Offline Model Builder (*d*).

REIMS is a destructive method, in which materials are combusted by a diathermy current, and the aerosol subsequently ionized to generate a mass spectrum. To test whether rapid evaporative ionization can generate informative mass spectra from insect samples, we conducted some initial investigations on five arthropods, the garden spider (Araneidae), the nettle aphid (Aphididae), the common wood louse (Oniscidae), a springtail (Collembola) and a damsel bug (Nabidae). For these samples, relatively small numbers of individuals were collected in the field and analysed. However, each species yielded detailed REIMS mass spectra, and the spectra were visually distinct from each other. Even with the caveat of small numbers, the five species were readily resolved by PCA and LDA of the ensuing mass spectra, clustering members of one species together and convincingly resolving different species (figure 1).

**Figure 2.**
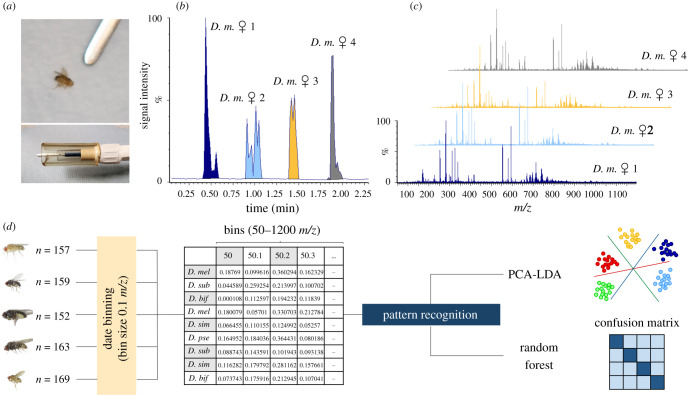
REIMS analysis of *Drosophila* species. *Drosophila* specimens were analysed using the electrosurgical pen with knife attachment, surrounded by a plastic tube to enhance capture of the aerosol (*a*). Each sample was completely consumed in a burn event that differed in shape and intensity for individual specimens (four individuals, *b*). The mass spectra from individuals was consistent, irrespective of shape or duration of the burn event (*c*). For subsequent data analysis the spectra were lock mass corrected, the background was subtracted, and the high-resolution mass spectra were compartmentalized to 0.1 *m/z* wide bins prior to further analysis (*d*). Abbreviation: D.m: *Drosophila melanogaster*.

Having established proof-of-concept data that arthropods were able to yield detailed REIMS spectra that could readily be used to discriminate species, we explored the subtlety of the method in a more closely focused study, based on a higher number of individuals from different laboratory-reared *Drosophila* species. Adult male and female *D. melanogaster*, *D. subobscura*, *D. pseudoobscura*, *D. bifasciata* and *D. simulans* were killed by freezing and stored at -20°C for several days before being analysed in a randomized order. The analysis was conducted in a similar fashion to the arthropods: the individuals were placed on wet glass fibre paper and aerosolized using an electrosurgical pen with knife attachment at a power level of 40 W. However, an additional wide piece of tubing (figure 2*a*) was used to maximize aerosol collection and ensure comparable aerosol aspiration among samples. The complete set-up is depicted in electronic supplementary material, figure S1. Analysis of a single fly (dry weight approx. 200 µg, bionumbers.hms.harvard.edu) generated sufficient aerosol to create a strong REIMS signal.

Replicated analysis of specimens, even from the same species and sex, can lead to the elaboration of different signal profiles over time (burn events) when expressed as a time-dependent total ion current (TIC) trace (figure 2*b*); this is because of variability in the manual position of a relatively large REIMS electrode on a small subject (figure 2*a*). However, the mass spectra, summed across the burn events, yielded consistent mass spectra (figure 2*c*) and data derived from different individuals were readily combined into one group or classification cluster. The first data processing step reduces the complexity of the mass spectral data by binning into 0.1 *m/z* wide windows. Registration and alignment of individual mass spectra are achieved by locking them, in a post-acquisition step, to the used ‘lock mass' (leu-enkephalin, at *m/z* 554.26), analysed continuously throughout sample analysis. The *m/z* data, aligned and binned, facilitated subsequent analysis and model building through pattern recognition algorithms, including PCA and LDA as well as random forest classification.

**Figure 3.**
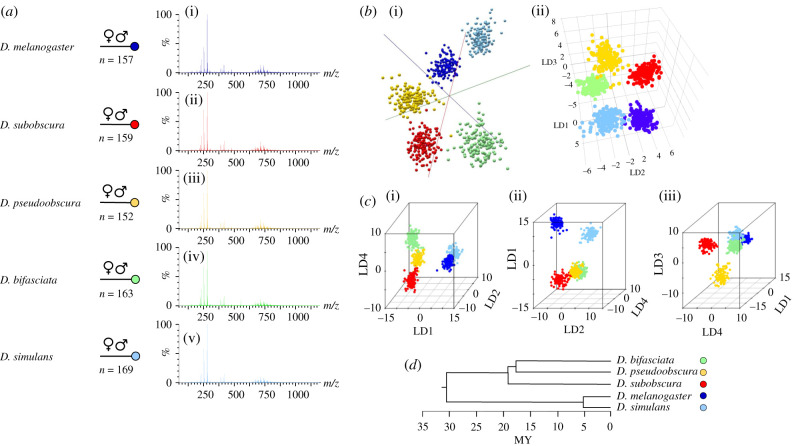
Species discrimination of *Drosophila* by REIMS. Five *Drosophila* species (800 samples in total) were analysed by REIMS. Representative mass spectra (of female specimens, males not shown) are given in *a*. The discretized, binned mass spectra were used to build the species discrimination model. REIMS data were analysed using the model building software packages Offline Model Builder (i) and LiveID (ii), both constructed the species separation model using PCA-LDA (*b*). Additionally, PCA-LDA separation was performed in R and visualized using different orientations and combination of linear discriminants (*c*). The clustering of the data points correlates with the phylogenetic relatedness of the five species (*d*).

The mass spectra originating from different *Drosophila* species exhibited an overall similarity (figure 3*a*), reducing the possibility of species-specific ions that would allow separation and identification. Due to the complexity and similarity of the REIMS spectra, data analysis was based on pattern recognition algorithms, which take into account the differences in overall mass spectral patterns rather than focus on differences in a single ion. This approach has the advantage that small differences in the abundance of specific ions between two groups can still be useful for separation purposes when combined with further differences elsewhere in the mass spectrum.

The mass spectra obtained from five species were imported to model building software packages LiveID and Offline Model Builder (both Waters) or divided into the five species classifications. The settings for data processing and model building used in each software are specified in the Methods section. The models, whether from LiveID and Offline Model Builder, yielded successful separation of the five *Drosophila* species using PCA and LDA. (Figure 3*b*)

The separation could be optimized by the number of principal components (PCs) chosen for LDA; more PCs means added information, but also variance is incorporated into the model. The models were adjusted individually to find the optimal number of PCs: 100 PCs were used in Offline Model Builder (maximum number), 500 PCs in LiveID and R. Separation was achieved with 100 PCs, additional variance (PCs) only served the purpose of fine tuning with modest added gains (example in electronic supplementary material, figure S2).

The separation between the classification groups in the models is uneven, placing *D. bifasciata*, *D. pseudoobscura* and *D. subobscura* closer but separated from a second group comprising *D. melanogaster* and *D. simulans*. This separation into groups of three and two species is especially pronounced in the PCA-LDA model created in R (figure 3*c*), due largely to differences in linear discriminant 1 which has the largest discriminatory power in the dataset (0.52). The results can be correlated with the phylogeny of the five species (figure 3*d*), which demonstrates similar clustering. Within each group, the member species are also differentiated. The separation of *D. melanogaster* and *D. simulans* highlights the ability of REIMS to distinguish even closely related species that are phenotypically distinguishable only by examining male genitalia. As females of *D. melanogaster* and *D. simulans* cannot reliably be distinguished phenotypically [52], a separate model was built only using the females of both species (electronic supplementary material, figure S3). The variance in the lipid/metabolite profile is greater between *D. melanogaster* and *D. simulans* than between the other three species (*D. subobscura*, *D. bifasciata* and *D. pseudoobscura*) as they can be resolved by linear discriminant 2 (0.24; figure 3*c* centre), while the larger group is resolved by linear discriminants 3 (0.15) and 4 (0.1) (figure 3*c*iii).

**Figure 4.**
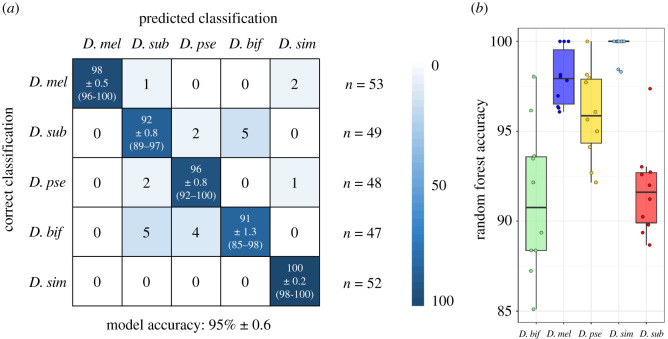
Classification of *Drosophila* species by random forest analysis. The binned *m/z* data from five species and both sexes were analysed by random forest analysis and repeated 10 times, using different randomly selected training (70% of the data) and test (30% of the data) datasets. (*a*) The confusion matrix contains the mean percentages of correctly identified and misidentified samples for every species, rounded to the nearest integer, as well as the standard error of the mean. The range of species classification accuracy for each of the 10 models (lowest and highest percentage) is listed in parentheses below the standard error of the mean. The average number of samples per species used for testing the model are listed on the side (*n* = x). The overall model accuracy was 95 ± 0.6% (mean ± SEM). For the 10 individual random forests, prediction accuracies for each species are plotted in *b* (median, 25th and 75th percentiles, all data shown). Abbreviations are *D. mel*: *Drosophila melanogaster*, *D. sub*: *Drosophila subobscura*, *D. pse*: *Drosophila pseudoobscura*, *D. bif*: *Drosophila bifasciata* and *D. sim*: *Drosophila simulans*.

In addition to PCA and LDA, the datasets were analysed using random forest classification. Here, the data were split before each analysis; 70% being used for model building, the remaining 30% were used to test the classification performance. For each model, random forest analysis was repeated 10 times, leading to different randomly selected datasets for training and testing every time. The number of trees used for forest calculation was chosen by comparing every possible number of trees between 1 and 2000 and their respective error rates (electronic supplementary material, figure S4). The number of trees used was the same for every repeated analysis. For species separation, the number of trees was set to 1500 and each forest was built and tested using the 70% model/30% test data. The classification performance is displayed as a confusion matrix of identification for all species (figure 4).

For every species, a correct classification rate (mean % ± SEM) of 91 ± 1.3 or higher was achieved, the overall model scored an accuracy of 95 ± 0.6. Thus, on average, 95 specimens out of 100 can be assigned to the correct species by employing REIMS data for model building, using only a few seconds of acquisition time for each insect. In the case of *D. simulans*, it is unlikely that samples would be mistaken for the closely related *D. melanogaster*, showing no difficulties in distinguishing even the females, despite their near-identical morphology.

**Figure 5.**
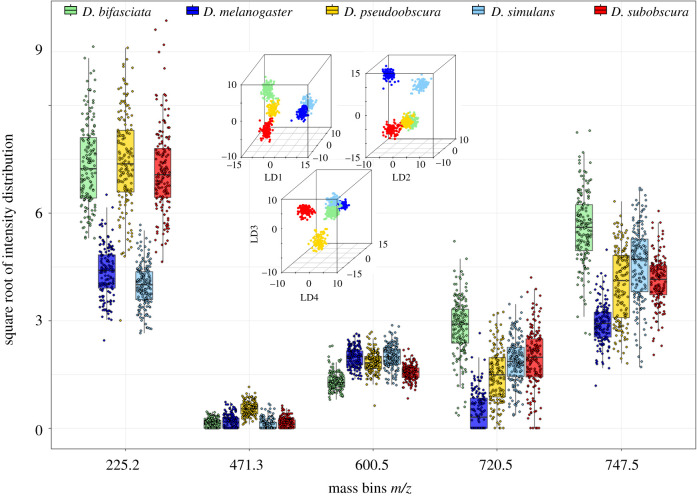
Comparative *m/z* bin intensities for five *Drosophila* species. The *m/z* bins that are most important for the resolution of five species by random forest were identified and their individual intensity values plotted here for every individual of each species (male and female samples are not discriminated). These *m/z* bins were repeatedly identified as essential separators for the random forest models, using the R package randomForestExplainer. The pattern within each bin shows its contribution to the identification process, highlighting the differences in relative abundance among the five *Drosophila* species.

Following random forest classification, another R package, randomForestExplainer [49], was used to extract information about the variables that contributed to class separation. In a top 10 approach, only variables that were registered as important in all repeated random forest runs were included. Additionally, the ^13^C isotopomers of certain variables were removed, after testing the pairs in question for correlation (electronic supplementary material, figure S5). To visualize how and to what extent the variables add to the separation of the five *Drosophila* species, the bin intensities were plotted (figure 5). The resulting intensity distribution of the top five variables allows interpretation of the relative molecule abundances and their impact on the classifying model.

The five most important variables for species resolution cover a fairly wide mass range, starting with the bin at *m/z* 225.2 ranging to the bin at *m/z* 747.5. The former might represent a fatty acid, whereas the latter is likely to be a phospholipid [37]. The ion bin at *m/z* 225.2 seems to define a major difference between the *D. melanogaster/D. simulans* group and the other species, which was already observed in the PCA-LDA models. The higher mass range bins, *m/z* 720.5 and *m/z* 747.5, display intensity variances that contribute to the discrimination of *D. melanogaster* and *D. simulans*. To distinguish *D. subobscura*, *D. bifasciata* and *D. pseudoobscura*, however, a combination of several ions with smaller variance is needed.

To confirm that the model separated species based on real rather than chance differences (given the large number of ion bins), the model was re-built using randomly assigned classification of each specimen to species. As expected, the model was incapable of separating species when spectra were randomly assigned. A comparison of the species models (built using the Offline Model Builder software), with correct and with randomly assigned classifications is presented in electronic supplementary material, figure S6. The results of the cross-validation performed after PCA-LDA (details are listed in the methods section) using Offline Model Builder and LiveID software are summarized in electronic supplementary material, figure S7.

### 3.1. Sex separation

**Figure 6.**
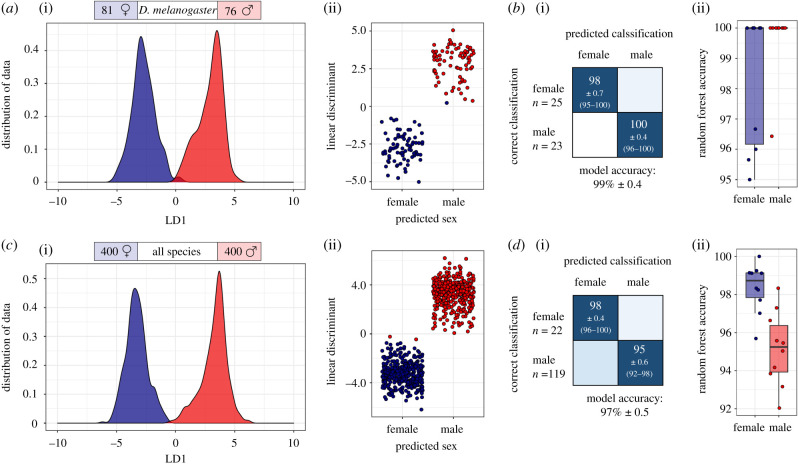
REIMS can discriminate sex. Separation of male (red) and female (blue) specimens of either *D. melanogaster* (*a*,*b*) or of all five species combined (*c*,*d*). The models were built using PCA-LDA, results are visualized in form of kernel density and scatterplots (*a* and *c*), or random forest analysis (confusion matrices and boxplots, *b* and *d*). The random forest models, built and tested 10 times each with a different 70%/30% training/test split, reached an average percentage accuracy of 99 ± 0.4 (mean ± SEM, *n* = 10, *D. melanogaster* only) and 97 ± 0.5 for all species. The boxplots on the right of the confusion matrices display the accuracies of all 10 random forest models for both classes, male and female.

The acquired REIMS data were used not only to discriminate species but were also investigated for its potential to distinguish sex. The sample analysis randomization was blind to species and to sex. Initially only *D. melanogaster* specimens were used for model building, to test if the REIMS spectra exhibited sex-specific variance of sufficient magnitude for separation (figure 6*a,b*; upper half). The average accuracy of the random forest classification (10 repeats) of males and females of *D. melanogaster* is 99 ± 0.4% (mean ± SEM), with only 2% of females misclassified as males and no males misclassified as females. PCA-LDA (using 80 PCs) yields a clear separation of male and female conspecifics, thus supports the existence of sex-specific variance in the REIMS spectra.

To further explore the ability to resolve sexes, independent of the species attribute, males and females of all five *Drosophila* species were combined for model building in a subsequent step. A resolving pattern, true for every species, reached 97 ± 0.5 (mean% ± SEM, *n* = 10) accuracy in random forest analysis, only 2% lower than the accuracy obtained with a single species. Both types of analysis, random forest and PCA-LDA, agree that only a few samples are confused in the classification process. (Figure 6*c*,*d*) Subsequently, samples were randomly assigned to the male or female category, anticipating a large overlap between the two classes in a repeated classification attempt. As expected, the classifications were substantially worse. A comparison of PCA-LDA separation with correctly and randomly assigned classifications for the *D. melanogaster* model, as well as for the model including all species, is presented in the electronic supplementary material, figures S8 and S9. In addition, both sex separation models were built with a lower number of PCs, proving that the numbers of PCs used in figure 6 were maximized for optimization, but not essential to achieve separation (electronic supplementary material, figures S10 and S11).

### 3.2. Species separation using *Drosophila* larvae

**Figure 7.**
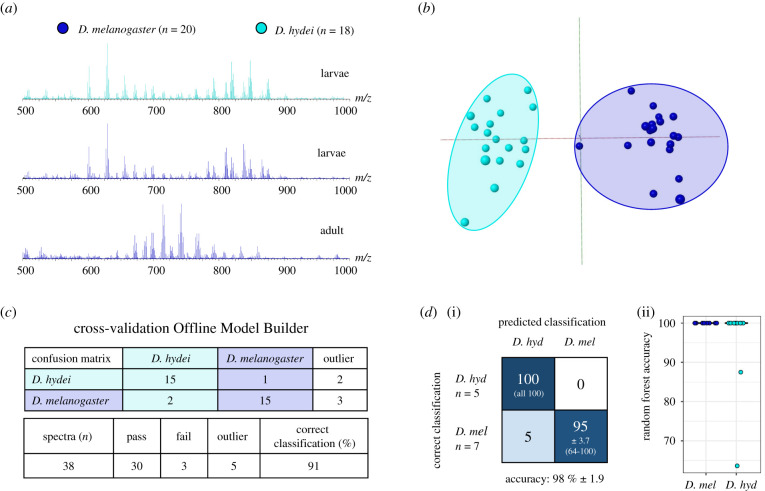
REIMS can discriminate species at the larval stage. Larvae from two *Drosophila* species (*D. melanogaster* and *D. hydei*) were analysed by REIMS. The mass spectrum obtained from the larval stage was clearly different to the adult, but both larval species yielded similar spectra (*a*) that permitted discrimination by PCA-LDA (*b*). Distinct discrimination between species was obtained through cross-validation in Offline Model Builder (*c*). The random forest models (*d*), built and tested 10 times each with a 70%/30% training/test split, reached an average percentage accuracy of 98 ± 1.9 (mean ± SEM, *n* = 10). The boxplot adjacent to the confusion matrix displays the performance for each species across all 10 random forests.

After successfully separating adult specimens of highly similar morphology (females of *D. melanogaster* and *D. simulans*), REIMS capabilities were further tested using a small set of *Drosophila* larvae. Larval *Drosophila* of closely related species are typically very difficult to identify, requiring skilled microdissection and morphological analysis under a microscope [53], with many species pairs being impossible to distinguish until adulthood [54]. For this preliminary experiment, the larvae of *D. melanogaster* and *D. hydei*, all in the 3rd instar stage, were analysed by the same procedures and settings as adult specimens. The REIMS spectra resulting from the two species in their larval stage are highly similar, but interestingly, exhibit a mass spectrum that is different from specimens in their mature state. Even if larvae and adult are derived from the same species, shown here for *D. melanogaster*, there is a substantial difference in the spectrum in the higher mass region (*m/z* 600–900; figure 7*a*)

Despite the observation that the mass spectra of the *D. melanogaster* and *D. hydei* larvae were strongly alike, the *m/z* bin data matrices were used to perform PCA-LDA and random forest analysis to explore species-related variance of larval samples. Despite the small number of samples, both types of analysis located sufficient differences in the mass patterns to provide a clear separation between the two species (figure 7*b*,*d*). To gauge the model's performance, cross-validation was carried out within Offline Model Builder (leaving 20% of data out). The results, including a confusion matrix, outlier numbers, as well as the correct classification rate, are presented in figure 7*c*. Random classification assignment, by contrast, led to considerable overlap between the two species (electronic supplementary material, figure S12).

These results suggest that REIMS could be used to identify insects, whether they are mature or in their immature developmental stages (photos of *Drosophila* adults in electronic supplementary material, figure S13). Even in cases of similar or near-identical morphology, a number of differences can be found in the REIMS profiles. Despite those differences being small and variable, pattern recognition across numerous differences facilitated consistent classification, and hence the separation of species and sex in this study. Without the need for sample preparation, entomological expertize or perfectly preserved specimens, REIMS with pre-built pattern recognition models could allow identification within seconds, offering a significant time advantage over other methods. Further investigation of the method's suitability and limitations, focused on identification and characterization of insects, is of course required. Factors such as feed, age of the specimens and storage conditions or length of storage can be expected to impact the pattern-based models to various degrees. In order to build a robust and reliable identification system, capable of identifying a wide array of specimen and independent of their inherent properties, these variables will need to be taken into account. The speed of data acquisition and the subtlety of discrimination are promising and advocate the exploration of REIMS as a new insect identification tool.

## Supplementary Material

Supplementary Material

## Supplementary Material

Video File 1

## Data accessibility

All raw data files are freely available in the MetaboLights database with the accession number MTBLS1878. Link: https://www.ebi.ac.uk/metabolights/MTBLS1878/descriptors
