# Supplementary material for: The application of rapid evaporative ionization mass spectrometry in the analysis of Drosophila species—a potential new tool in entomology
Source: Open Biol. 2020 Nov 25;10(11):200196. doi: 10.1098/rsob.200196 (PMC7729031; doi:10.1098/rsob.200196)
Supplement: Supplementary Material [file rsob200196supp1.pdf]

The application of rapid evaporative ionization mass spectrometry in the analysis of *Drosophila* species—a potential new tool in entomology

Iris Wagner[1], Natalie I. Koch[1], Joscelyn Sarsby[1], Nicola White[2], Tom A. R. Price[2], Sam Jones[3], Jane L. Hurst[4], Robert J. Beynon[1,*]

Supplementary Figures

Supplemental Figure 1:
Overview of the REIMS set-up showing an instrument schematic (Waters, REIMS Research System with iKnife Sampling) (a), the actual lab set-up (b), a close-up of the electrosurgical pen with knife attachment (c) and additional wide tubing, which was used to increase aerosol uptake (d). In addition, a short video was filmed (included as a separate file, ***video file 1****) showing the process of sample burning and real-time analysis of the aerosol and signal acquisition in the mass spectrometer (e).*

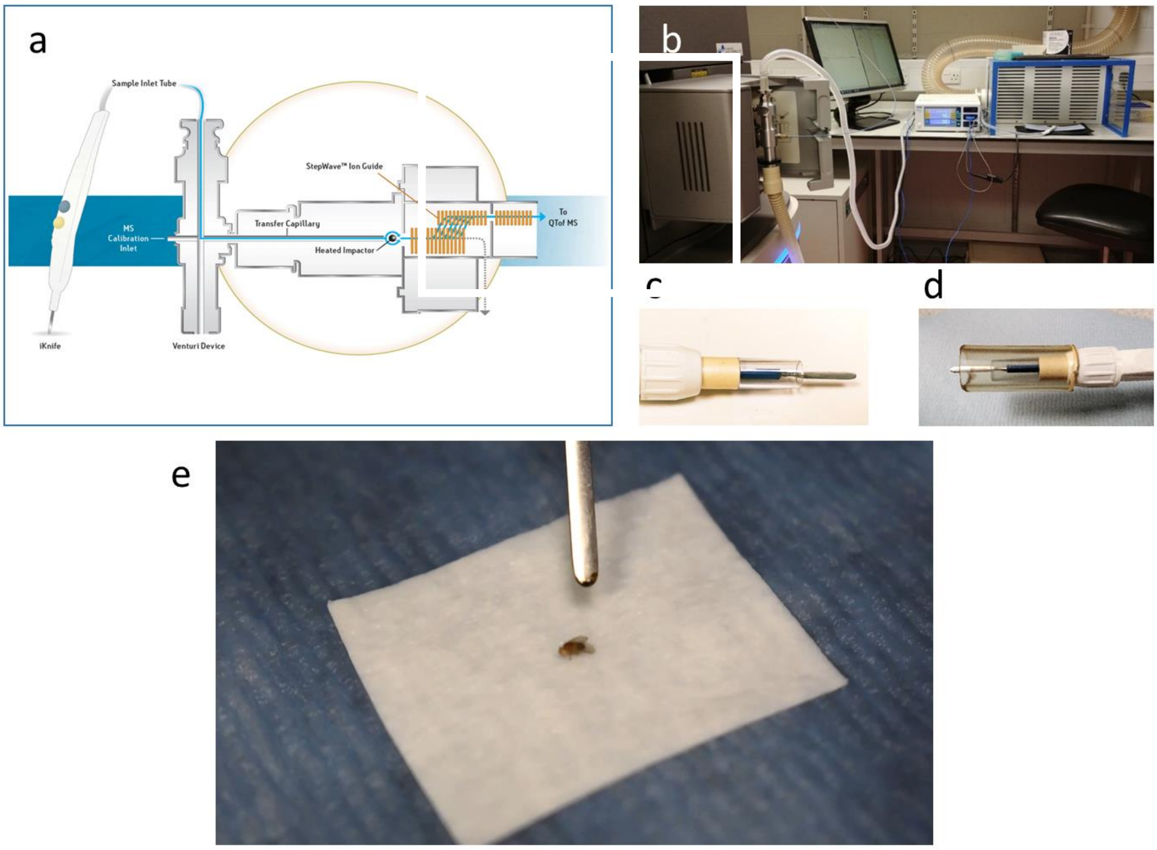

Supplemental Figure 2:

Comparison of the species separation achieved through PCA-LDA using 500 and 100 principal components. A clear clustering of samples into species classifications can already be observed using only 100 components, an increase to 500 components merely served the purpose of fine-tuning to optimise separation.

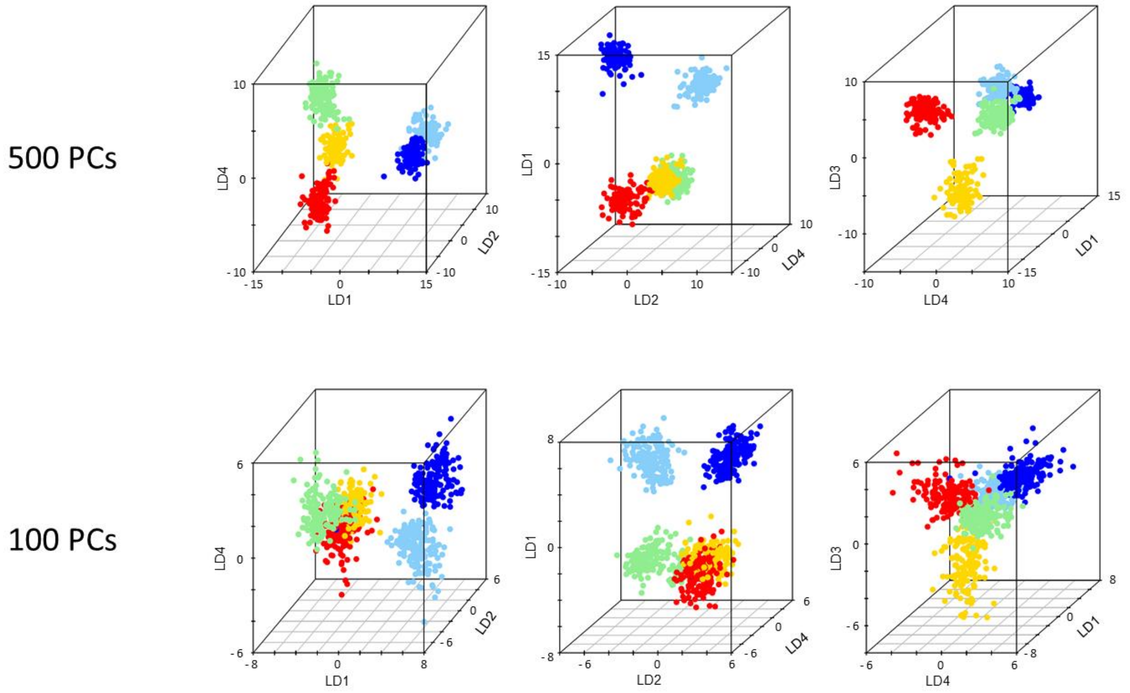

Supplemental Figure 3:

Separation of D. melanogaster *and* D. simulans *using only the morphologically highly similar females. Pictures of the flies and the number of samples used for model building are listed under (a). The two species were successfully separated using PC-LD analysis (in Offline Model Builder)(b). A model based on randomly assigned classifications did not yield a separation (c).*

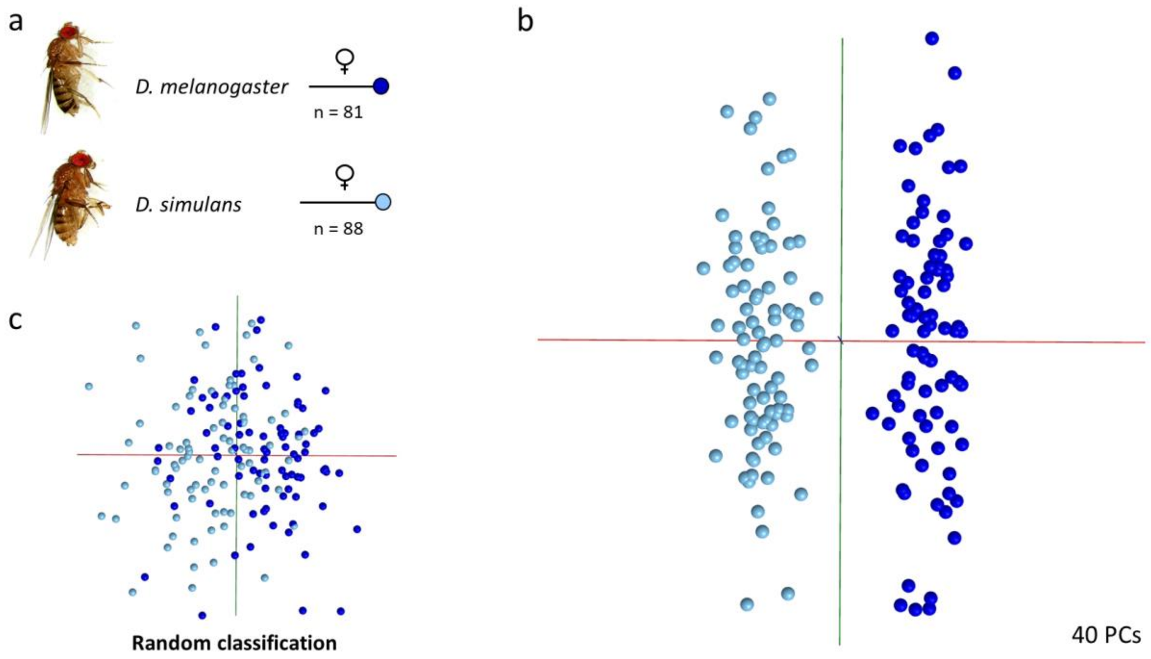

Supplemental Figure 4:

Random forest errors plotted against tree number (up to 2000 trees) for the presented models. Selecting the number of trees used for Random Forest analysis was based on error rate and stability of the error rate over a range of tree numbers. The best mtry was established during the first random forest analysis of each model and, like the tree number, kept the same for every repeat.

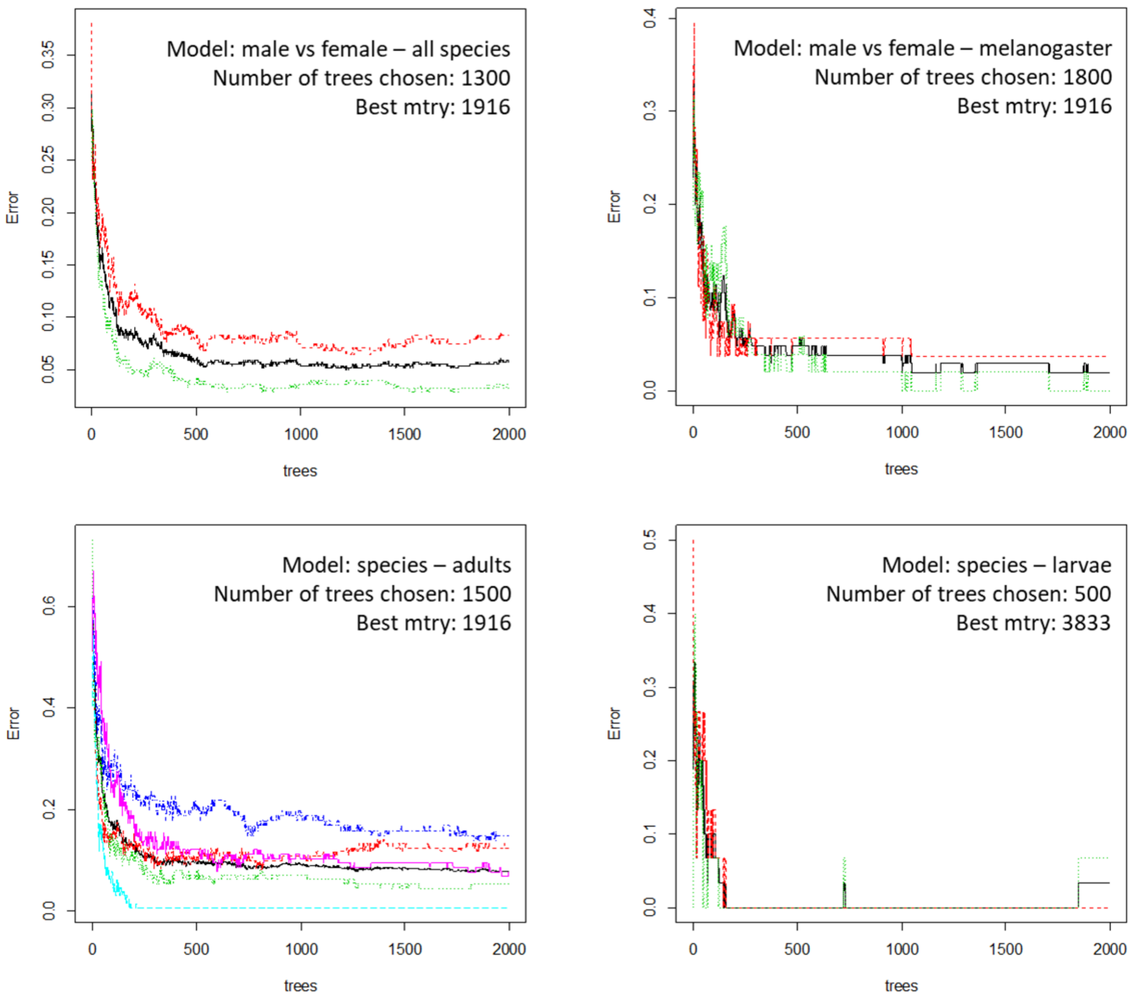

Supplemental Figure 5:

The top 10 variables influencing the separation of the five Drosophila species, identified using random forest explainer, contained potential isotopomers. The mass bins in question are directly compared and their intensities (in all 800 samples) correlate enough to confirm their status as isotopomers. As expected, they also contribute to the species separation in the same way, which might emphasise the variables importance, but renders them redundant in the separation process.

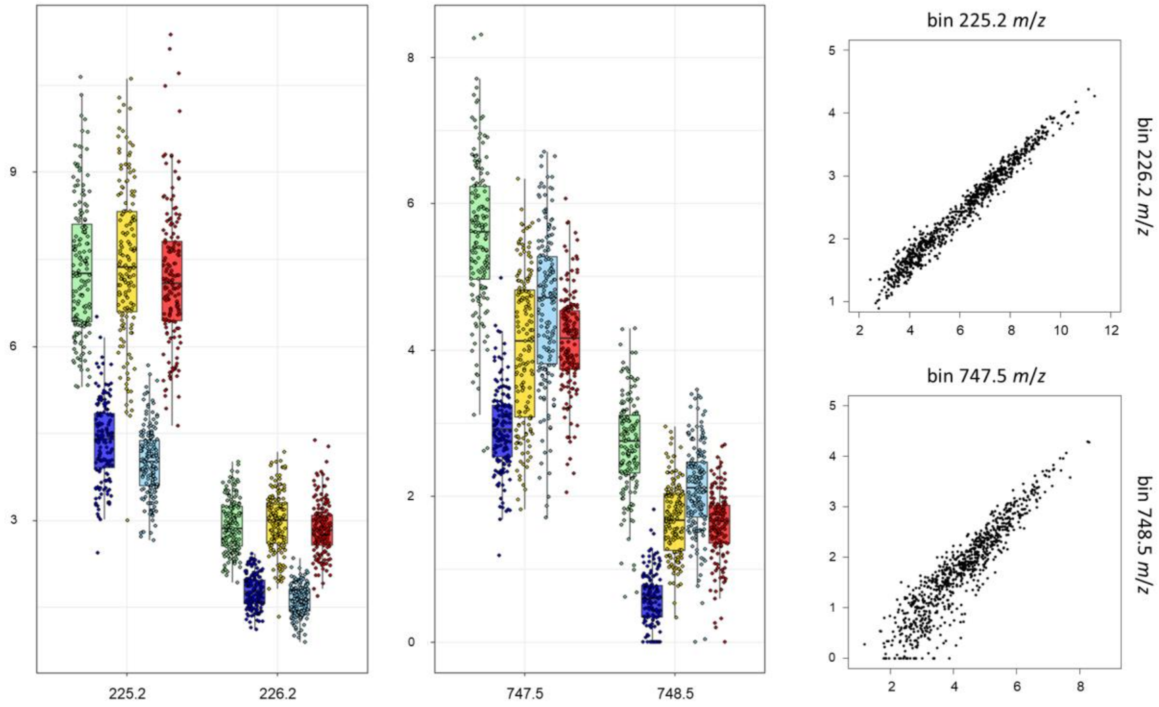

Supplemental Figure 6:

Comparison of PCA-LDA results using correctly and randomly assigned species classifications. A clear difference can be found when the species information is correct. When the five classifications are randomly assigned to samples, groups completely overlap and no distinction can be found, proving that the successful separation, of correctly assigned classifications, is based on species-specific variances.

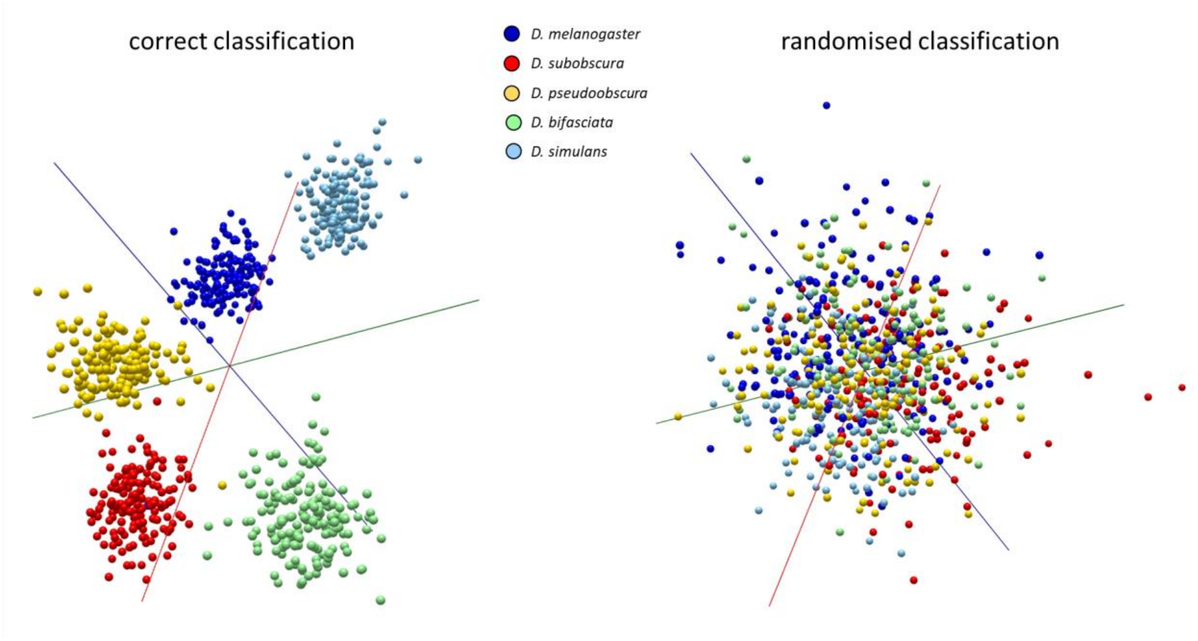

Supplemental Figure 7:

The PCA-LDA based species models were cross-validated within Offline Model Builder and LiveID, using the setting 'Leave 20 % out' and a standard deviation of 5.

a

Cross-validation *Offline Model Builder*:

Confusion matrix	*D. melanogaster*	*D. subobscura*	*D. pseudoobscura*	*D. bifasciata*	*D. simulans*	Outlier
D. melanogaster	155	1	0	0	0	1
D. subobscura	0	158	1	0	0	0
D. pseudoobscura	0	1	150	1	0	0
D. bifasciata	0	1	0	162	0	0
D. simulans	0	0	0	0	168	1

Spectra (n)	Pass	Fail	Outlier	Correct Classification (%)
800	793	5	2	99.37

b

Cross-validation *LiveID*:

Confusion matrix	*D. melanogaster*	*D. subobscura*	*D. pseudoobscura*	*D. bifasciata*	*D. simulans*	Outlier
D. melanogaster	154	1	0	0	0	2
D. subobscura	0	157	1	0	0	1
D. pseudoobscura	0	0	149	1	0	2
D. bifasciata	0	0	0	160	0	3
D. simulans	0	0	0	0	168	1

Spectra (n)	Pass	Fail	Outlier	Correct Classification (%)
800	788	3	9	98.50

Supplemental Figure 8:

Comparison of PCA-LDA separation of males and females of Drosophila melanogaster *using correct and randomly assigned classifications. Both separations are based on 80 principal components.*

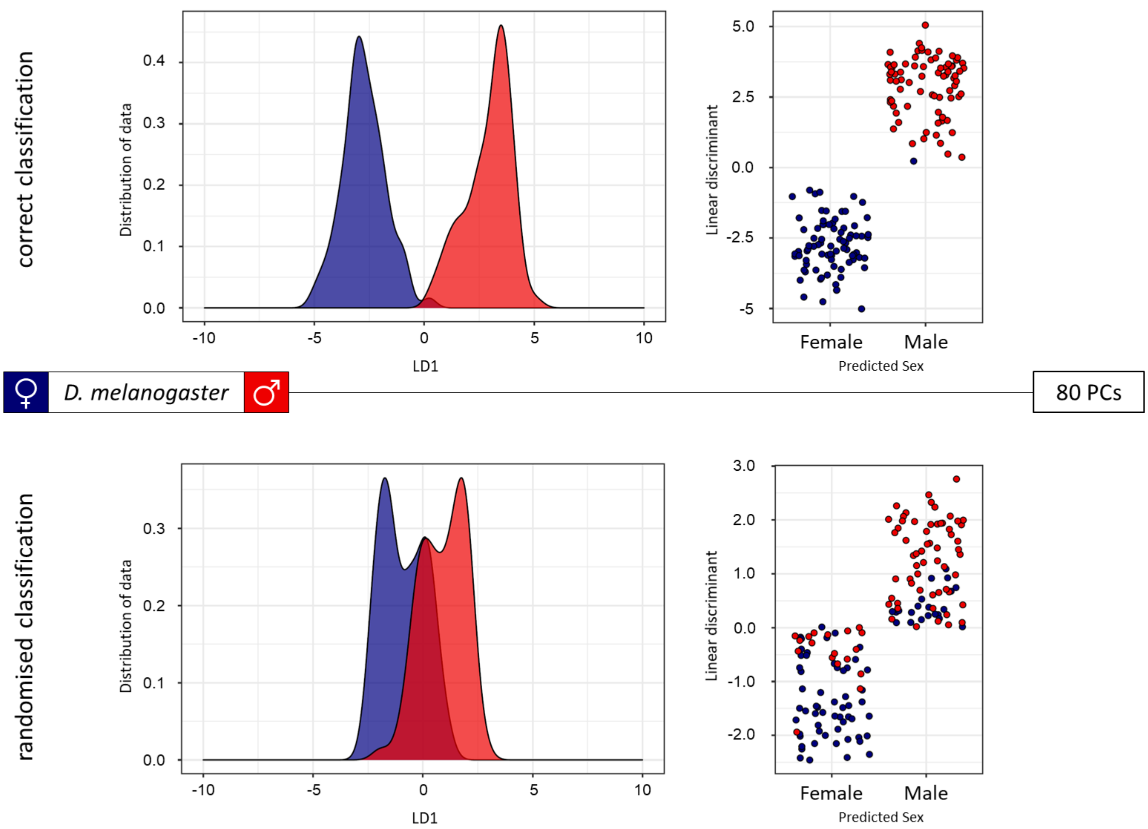

Supplemental Figure 9:

Comparison of PCA-LDA separation of males and females of all five species using correct and randomly assigned classifications. Both separations are based on 400 principal components.

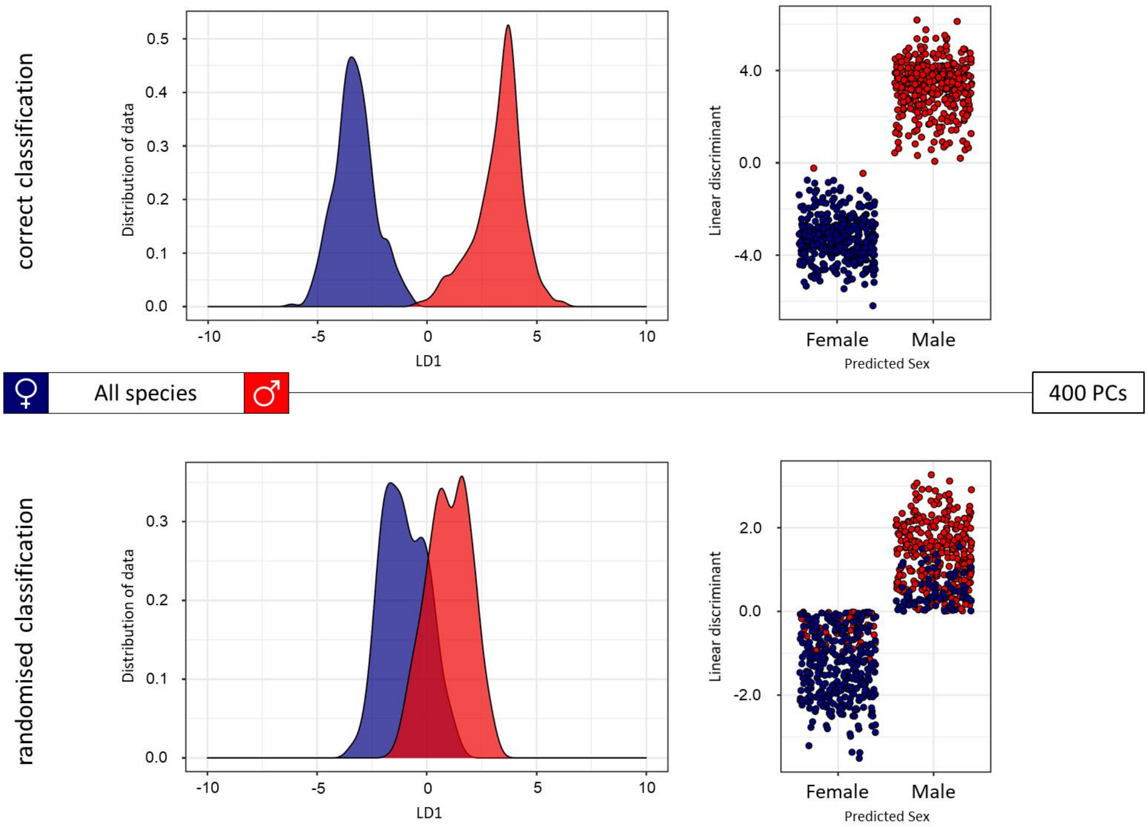

Supplemental Figure 10:

Comparison of PCA-LDA separation of males and females of Drosophila melanogaster *using correct and randomly assigned classifications. Both separations are based on 40 principal components, a quarter of the maximum number of components possible. Despite the lower number, males and females are separated when using the correct assignment of classes and overlap when samples are randomly assigned to a sex category.*

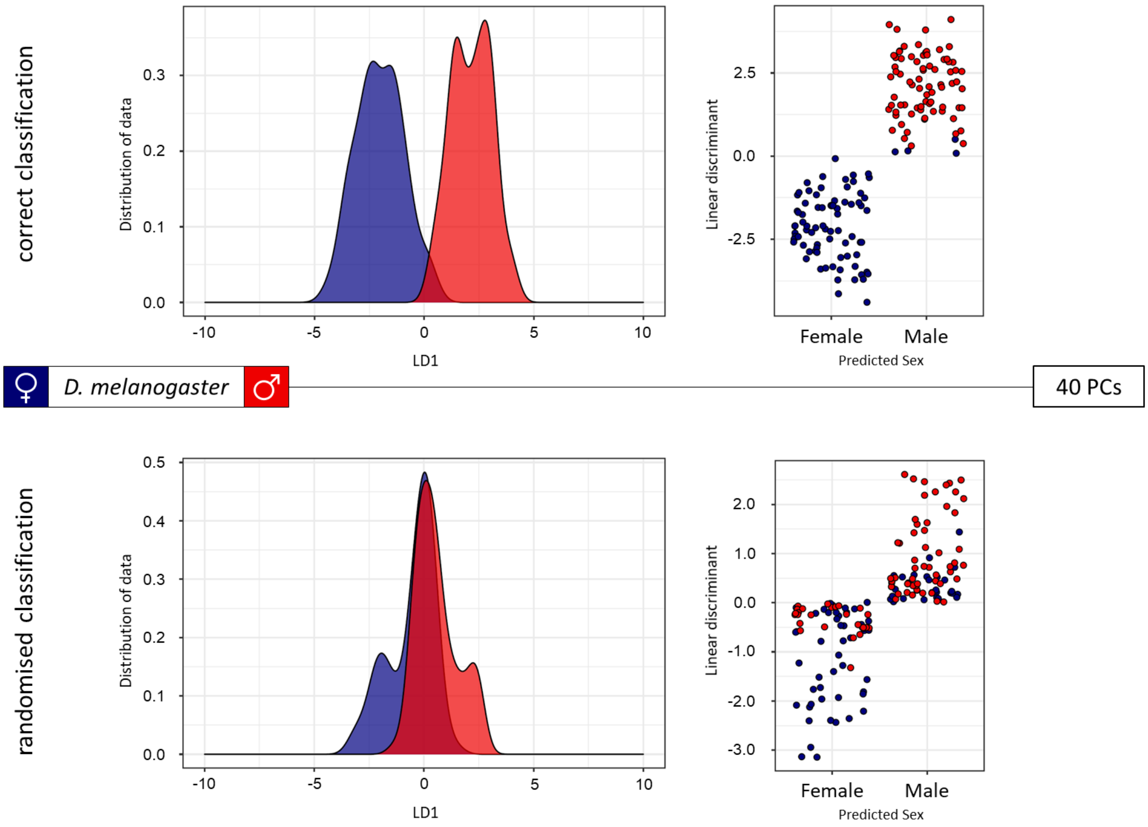

Supplemental Figure 11:

Comparison of PCA-LDA separation of males and females of all species using correct and randomly assigned classifications. Both separations are based on 200 principal components, a quarter of the maximum number of components possible. Despite the lower number, males and females are separated when using the correct assignment of classes and overlap when samples are randomly assigned to a sex category.

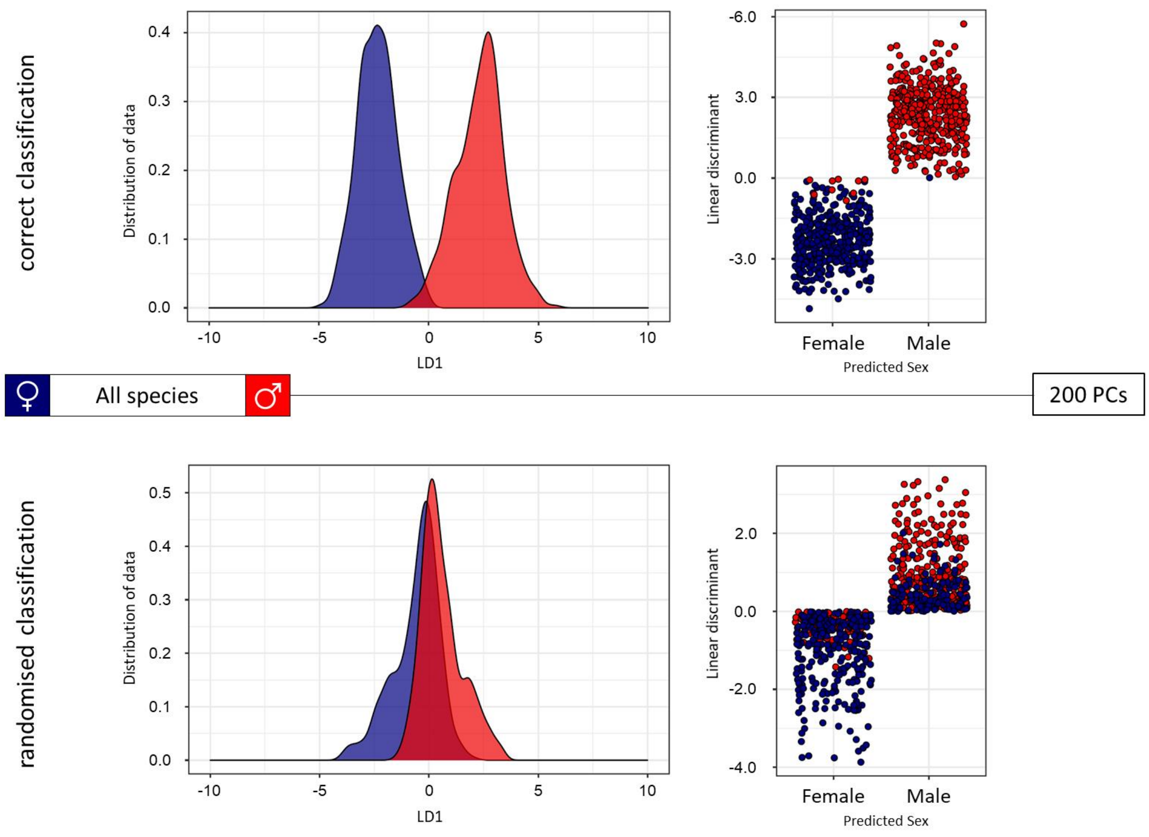

Supplemental Figure 12:

Comparison of PCA-LDA separations of D. melanogaster *and* D. hydei *larvae achieved when assigning samples the correct or a random classification. Due to small sample numbers of only two classifications, a full overlap in the randomised model is not expected.*

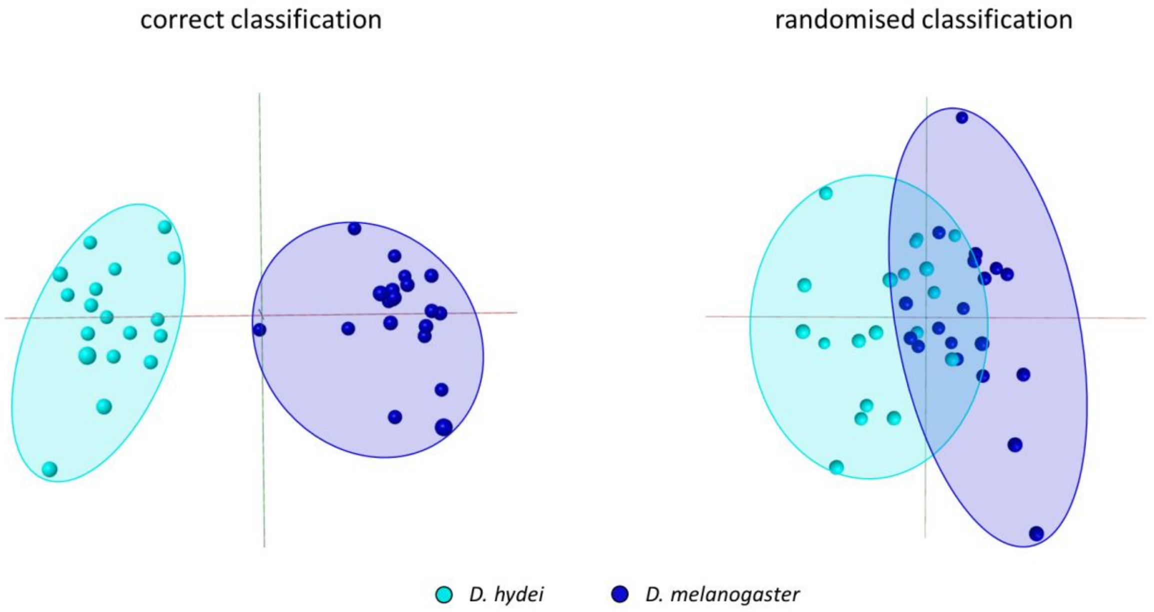

Supplemental Figure 13:

Photos of female specimens of the five Drosophila *species used in this study.*

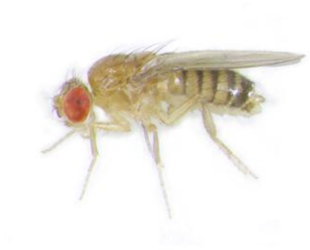

D. melanogaster

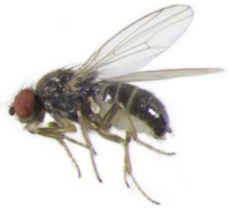

D. subobscura

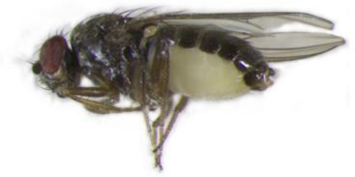

D. pseudoobscura

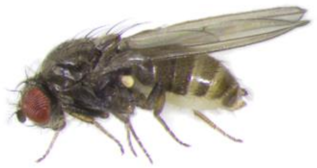

D. bifasciata

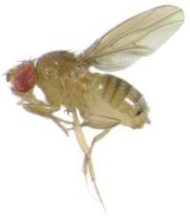

D. simulans
